# Molecular Targets of Cervical Cancer and Its Microenvironment: Advances in Treatment

**DOI:** 10.3390/cancers18040563

**Published:** 2026-02-09

**Authors:** Joe Youssef, Amal El Masri, Maya Atwi, Elio Ibrahim, Zahraa Salhab, Mohamad Badawi, Fatima Nazar, Jad El Masri, Wassim Abou-Kheir

**Affiliations:** 1 Department of Anatomy, Cell Biology and Physiological Sciences, American University of Beirut, Beirut 1107-2020, Lebanon; axm09@mail.aub.edu (A.E.M.); mya43@mail.aub.edu (M.A.); zs83@aub.edu.lb (Z.S.); mkb30@mail.aub.edu (M.B.); fan12@mail.aub.edu (F.N.); jse20@mail.aub.edu (J.E.M.); 2 Faculty of Medicine, Saint-Joseph University, Beirut 1104-2020, Lebanon; elio.ibrahim@net.usj.edu.lb

**Keywords:** cervical cancer, pathophysiology, targeted therapy, immunotherapy, therapeutic vaccines

## Abstract

Cervical cancer remains one of the most commonly diagnosed cancers in women worldwide, with an elevated number of associated annual deaths, even though effective preventive vaccines are available. The pathophysiology of cervical cancer is well understood, with high-risk HPV as the main culprit in carcinogenesis, in addition to cell proliferation signaling alteration and tumor immune microenvironment modulation. This understanding of the disease’s molecular signatures has led to the development of several lines of treatment, especially for advanced, recurrent, persistent, or metastatic disease. For instance, Bevacizumab, a monoclonal antibody targeting angiogenesis factors, as well as Endostar, a recombinant human endostatin, have been studied and shown to improve survival in advanced disease. In contrast, anti-angiogenic Tyrosine Kinase Inhibitors had limited efficacy. Furthermore, antibody-drug conjugates such as Tisotumab Vedotin allow to deliver a highly toxic payload directly to the tumor site by binding to tissue factor, which is highly expressed in cervical tumor cells. Moreover, immunotherapy has emerged as a key treatment modality in cervical cancer by inhibiting immune checkpoint inhibitors (PD1, PD-L1, and CTLA4). In addition, therapeutic vaccines have been investigated for the treatment of localized disease by enhancing cell-mediated immunity against E6 and E7 proteins. However, more robust clinical trials are needed before these vaccines can be effectively and safely used clinically. Finally, several ongoing trials are currently evaluating new therapeutic modalities and combinations of the currently available tools in the cervical cancer treatment armamentarium.

## Simple Summary

Cervical cancer remains a frequently diagnosed cancer among women and a major cause of death, despite efforts to reduce its occurrence with the preventive vaccines. Thanks to a better understanding of the molecular processes that define cervical cancer, newer approaches to tackle this cancer have been developed to supplement the traditional surgery, chemotherapy, and radiation. These include drugs that target blood vessel growth, specific antibodies that deliver a toxic drug directly to the cancer site, and immunotherapy that mounts the body’s immune system against the cancer cells. In addition, therapeutic vaccines that can be administered after the development of local lesions are being investigated as an additional treatment possibility. This review aims to summarize these emerging therapies and ongoing clinical trials, highlighting advances in cervical cancer treatment.

## 1. Introduction

Cervical cancer is a major global health concern for women, ranking as the fourth most frequently diagnosed cancer after breast, colorectal, and lung cancer [1]. In 2022, it was the most commonly diagnosed cancer and the primary cause of cancer-related death in 25 and 37 countries, respectively [2]. GLOBOCAN 2022 reported 662,301 new cases and 348,874 deaths globally [2]. By 2025, these figures were estimated to have increased to approximately 703,000 new cases and 373,000 related deaths worldwide [3]. This shows a concerning upward trend in the global burden of cervical cancer. By 2030, the yearly incidence of cervical cancer is expected to rise to nearly 700,000, while annual deaths are projected to reach 400,000 [4].

These global patterns prompt a closer look at how the burden of cervical cancer is distributed worldwide. Cervical cancer prevalence is closely linked to socioeconomic conditions, and in many low-income countries, it remains the primary cause of cancer-related mortality [5]. Approximately 85% of new cervical cancer cases and 90% of related deaths occur in low and middle-income countries [1]. This highlights the persistent disparities in prevention, early detection, and access to treatment across the world.

At the core of this global burden is human papillomaviruses (HPV), the primary causative agent of cervical cancer [2]. Infection with high-risk HPV alone, however, is not sufficient for malignant transformation. Other contributing risk factors include smoking, long-term oral contraceptive use, high parity, and sexually transmitted infections, particularly Chlamydia trachomatis and Human Immunodeficiency Virus (HIV) [2,6]. In 2020, about 5.8% of cervical cancer diagnoses worldwide occurred in women with HIV, and 4.9% of new cases were directly attributed to HIV infection [6].

To guide clinical decision-making, cervical cancer is classified into four main stages using the FIGO system, established by the International Federation of Gynecology and Obstetrics. In 2018, FIGO updated its staging system to incorporate available clinical examination, imaging, and pathology to determine disease stage [1]. This system categorizes the disease based on the extent of tumor spread and includes additional subdivisions for greater precision. It now includes 14 subgroups [5]. Stage I is confined to the cervix. As the cancer progresses to stage II, it extends beyond the cervix but has not yet reached the pelvic wall or the lower third of the vagina. Stage III reflects a more advanced spread, involving the lower third of the vagina, the pelvic wall, or the pelvic/para-aortic lymph nodes. Stage IV is the most advanced stage, marked by invasion of nearby organs or distant metastases [5].

The primary prevention measure against cervical cancer is HPV vaccination [1]. Several prophylactic vaccines (bivalent, quadrivalent, and nonavalent) are globally used to protect against high-risk HPV infections that cause precancerous lesions. Secondary prevention relies on screening programs that detect these high-grade precancerous lesions, such as CIN (cervical intraepithelial neoplasia) and AIS (adenocarcinoma in situ), allowing for early diagnosis and treatment before lesions progress to invasive disease. Because HPV vaccination and HPV-based screening are highly effective, cervical cancer is considered a preventable disease [7]. If detected early, cervical cancer can often be cured with surgery alone. However, in many low and middle-income countries, patients typically present at more advanced stages requiring radiation and chemotherapy due to limited access to screening and early detection programs [4].

This review focuses on recent advances in the treatment of metastatic and recurrent cervical cancer, emphasizing targeted, immune, and vaccine-based therapies (See Table 1). It also explores how emerging molecular insights and biomarkers guide therapeutic strategies.

## 2. Methods

A comprehensive search of the literature was conducted through electronic databases, including PubMed, Ovid Medline, and Cochrane Library, to identify relevant articles on novel therapeutic modalities for cervical cancer. The latest search was conducted on 18 January 2026. We use expanded Meshes and keywords including “Uterine Cervical Neoplasms”, “Angiogenesis Inhibitors”, “Bevacizumab”, Immunoconjugates”, “Immunotherapy”, “Vaccines”, using adjacency and truncation parameters to increase sensitivity. Articles were first screened by title and abstract, and full texts were retrieved for studies meeting our inclusion criteria. We included clinical trials, meta-analyses and translational studies involving women with cervical cancer that evaluated targeted therapy, immunotherapy, and therapeutic vaccines. We excluded non-English articles and case reports. Primary and secondary outcomes from phase I, II, and III trials were extracted by three different authors. We also looked at ongoing trials through Clinicaltrials.gov (https://clinicaltrials.gov/, accessed on 26 November 2025) trials looking at promising new therapeutic modalities or combination therapy.

## 3. Pathophysiology of Cervical Cancer

### 3.1. Human Papillomavirus

#### 3.1.1. Human Papillomavirus Oncoproteins

Persistent infection with high-risk HPV is the central initiating event in cervical carcinogenesis. The majority of cervical cancers are attributable to HPV, with HPV-16 and HPV-18 being the most carcinogenic and responsible for most cases worldwide [33,34]. The transforming capacity of HPV is mediated primarily through the viral oncoproteins E6 and E7. E6 binds p53 and promotes its ubiquitination and degradation, thereby preventing apoptosis and removing a critical checkpoint against genomic instability [33,35,36]. E7 targets pRb, functionally inactivating it and releasing E2F transcription factors that drive unrestrained transition from G1 to S phase and sustained proliferation [37,38]. In addition to E6/E7, HPV E5 contributes to immune escape by impairing MHC trafficking and antigen presentation, facilitating viral persistence, and allowing progressive malignant evolution [33,39,40]. Beyond direct cell-cycle deregulation, HPV infection also promotes oxidative stress and microRNA dysregulation, both of which further contribute to cervical transformation and progression [33,41]. In addition, HPV-driven oncogenesis appears to promote early angiogenic remodeling: E6/E7 expression disrupts the balance of pro- and anti-angiogenic factors and correlates with rising VEGF levels and vascular density across the dysplasia–carcinoma continuum [42,43,44].

#### 3.1.2. Human Papillomavirus Related and Unrelated Subtypes

Cervical cancer is currently classified into HPV-associated and HPV-unrelated entities based on distinct morphologic, molecular, and clinical characteristics. Most cervical squamous cell carcinomas (SCCs) are HPV-related and most commonly exhibit a non-keratinizing morphology, characterized by sheets or nests of basaloid cells with indistinct borders, high mitotic activity, minimal stromal response, and limited squamous maturation. In contrast, keratinizing SCCs, which demonstrate polygonal cells with abundant eosinophilic cytoplasm, prominent keratin pearl formation, and desmoplastic stroma, are more frequently associated with HPV-unrelated pathways, although overlap exists. Hybrid tumors showing both non-keratinizing features with focal maturation retain a strong association with HPV and should be distinguished from true keratinizing SCCs to avoid prognostic misinterpretation [45]. Among adenocarcinomas, the usual-type adenocarcinoma represents the prototypical HPV-associated glandular tumor and typically shows diffuse p16 overexpression as a surrogate marker of transcriptionally active HPV [46].

In contrast, HPV-unrelated cervical cancers constitute a biologically distinct subgroup, particularly enriched among adenocarcinomas. The most clinically relevant HPV-independent subtypes include gastric-type adenocarcinoma and clear cell carcinoma. Gastric-type adenocarcinoma is characterized by abundant pale or eosinophilic cytoplasm, deep stromal invasion, absent or patchy p16 expression, and frequent TP53 mutations, and is associated with aggressive behavior and poor response to conventional chemoradiation [47,48]. Clear cell carcinoma, marked by clear or hobnail cells and expression of markers such as HNF1β and Napsin A, is also HPV-independent and often presents at advanced stages, with limited sensitivity to standard therapies [49,50]. Overall, HPV-unrelated tumors lack viral oncogene-driven carcinogenesis, display distinct molecular alterations, and are associated with worse prognosis compared with HPV-associated counterparts, which thus underscores their emerging clinical and therapeutic significance [46,51].

### 3.2. Signaling Pathways

#### 3.2.1. Telomerase Activity

Telomerase activation plays a key role in malignant progression. Studies have found that increased expression of TPP1, which recruits telomerase and supports telomere elongation, correlates with rising hTERT expression across premalignant stages and invasive disease [52,53,54]. Both high TPP1 and co-high TPP1/hTERT expression independently predict worse survival [55]. These findings prove that telomerase upregulation is an early and prognostically meaningful driver of cervical cancer.

#### 3.2.2. PI3K/AKT/mTOR Axis

In parallel, several oncogenic signaling pathways become progressively activated. The PI3K/AKT/mTOR axis is frequently dysregulated in cervical cancer, with overexpression of PI3K in tumor lines and activated AKT/mTOR correlating with poorer outcomes, chemoresistance, and inferior response to radiotherapy [56,57,58]. Early clinical trials with mTOR inhibition, including temsirolimus, show limited but real disease-stabilizing activity, highlighting the pathway’s significance as a target for therapy [59].

#### 3.2.3. Wnt/β-Catenin Axis

Concurrently, canonical Wnt/β-catenin signaling is commonly activated in cervical carcinoma. Shinohara et al. have found that increased cytoplasmic and nuclear β-catenin is observed in most invasive tumors despite infrequent β-catenin mutations, thus suggesting activation through upstream regulation. This could potentially involve promoter methylation of Wnt antagonists or altered degradation complexes [60,61]. This Wnt activation supports transformation, invasion, and later epithelial–mesenchymal transition, and integrates with broader metastatic programs described in cervical progression models [61].

#### 3.2.4. Current Therapeutic Landscape

When considering these pathways from a translational perspective, they differ markedly in current clinical actionability. Among them, dysregulation of the PI3K/AKT/mTOR axis is the most therapeutically actionable in cervical cancer, given the availability of pathway-specific inhibitors and early clinical evidence of disease stabilization with mTOR-targeted therapies [56,58]. In contrast, telomerase activation, while strongly linked to disease progression and prognosis, remains largely investigational as a direct therapeutic target [54]. Similarly, aberrant Wnt/β-catenin signaling, despite its central role in invasion and metastatic programs, presents significant challenges for safe and effective targeting and is not yet readily actionable in routine clinical practice [60,61]. This highlights the PI3K/AKT/mTOR pathway as the most promising target for near-term therapy.

### 3.3. Genomic Landscape: Three Distinct Types

Large integrative genomic studies identify significant molecular variability in cervical cancer. Multi-omic clustering identifies three biologically distinct subtypes: a squamous keratin-high cluster, a squamous keratin-low cluster, and an adenocarcinoma-rich cluster [62]. These groups largely correspond to histologic patterns but are further defined by differences in gene expression programs, HPV associations, and mutation profiles. Keratin-high squamous cancers show strong epithelial differentiation and keratinization, whereas keratin-low squamous tumors demonstrate reduced keratin gene expression and differential expression of pathways involving ARID1A, NFE2L2, and PIK3CA [63,64]. The adenocarcinoma-rich cluster includes most glandular tumors and contains a subset of HPV-negative, endometrial-like cancers enriched for mutations such as KRAS, ARID1A, and PTEN, which indicates distinct oncogenic dependencies [62]. Importantly, a majority of cervical cancers harbor alterations in PI3K–MAPK and TGF-β signaling, linking subtype-specific biology to pathways suitable for targeted intervention [62,65]. These genomic subtypes, therefore, offer a framework for understanding different tumor behavior and treatment response.

### 3.4. Tumor Immune Microenvironment

Cervical cancer develops in a uniquely virus-conditioned immune context, and epidemiologic evidence indicates that immune competence plays a central role in HPV clearance. Rates of cervical cancer and other HPV-associated malignancies are markedly increased in immunocompromised patients, including those with HIV/AIDS and solid-organ transplantation, supporting host immunity as a major determinant of carcinogenic progression [39,66]. Within tumors, HPV-antigen–specific T cells frequently infiltrate lesions, yet they often fail to eradicate disease because HPV-positive cancers enforce multiple immune-evasion strategies [39]. For instance, HPV E5 impairs antigen presentation by disrupting MHC transport, while E7 and E6 dampen innate antiviral signaling via the cGAS–STING pathway, collectively reducing interferon-mediated immune activation [67,68]. Cervical tumors also exhibit increased PD-L1 expression, in part directly induced by E7, which promotes T-cell dysfunction and exhaustion [69]. Furthermore, regulatory T-cell enrichment and systemic immune tolerance are observed across CIN and invasive disease, suggesting that immune suppression is both local and systemic [70]. Together, these features define an immunosuppressive tumor microenvironment that permits persistent HPV infection and supports malignant progression.

#### 3.4.1. Stromal Fibroblasts

Stromal fibroblasts actively contribute to cervical cancer progression by shaping a protumorigenic immune microenvironment and remodeling the tumor stroma. Cervical cancer cells instruct fibroblasts through paracrine IL-6 signaling, leading to C/EBPβ-dependent induction of the chemokine CCL20, which promotes recruitment of CCR6^+^ Th17 cells into the tumor stroma [71]. Predominant stromal CCL20 expression correlates with increased Th17 infiltration, advanced FIGO stage, and disease severity, supporting a chronic inflammatory milieu associated with poor prognosis [72]. Beyond recruitment, cancer-instructed fibroblasts secrete IL-6 and IL-1β, which enhance IL-23 production by myeloid dendritic cells while suppressing IL-12, thereby favoring Th17 expansion over antitumor Th1 responses [73]. In parallel, cervical cancer cells promote fibroblast activation through exosome-mediated transfer of Wnt2B, triggering Wnt/β-catenin signaling and driving the conversion of normal fibroblasts into cancer-associated fibroblasts (CAFs) with enhanced proliferative and migratory capacity [74]. Together, these mechanisms establish a self-reinforcing stromal–immune network that facilitates immune evasion, angiogenesis, and cervical cancer progression.

#### 3.4.2. Myeloid Populations

Myeloid cells are central regulators of the cervical cancer tumor microenvironment, linking HPV-driven chronic inflammation to immune evasion, stromal remodeling, and invasion. Tumor-associated neutrophils (TANs) can promote angiogenesis and invasion through secretion of VEGF and proteases such as MMP-2/MMP-9, which remodel the extracellular matrix and facilitate tumor cell migration; formation of neutrophil extracellular traps (NETs) may further amplify local inflammation and support metastatic potential, and higher neutrophil activity is frequently associated with worse outcomes [75]. In parallel, single-cell and spatial transcriptomic studies highlight the heterogeneity of tumor-associated macrophages and identify SPP1^+^ (osteopontin-high) macrophages as a prominent immunosuppressive subset in cervical cancer, particularly in HPV-positive tumors [76,77]. These macrophages exhibit transcriptional programs that impair antigen presentation and promote tumor survival, and SPP1–CD44 signaling has been implicated in T-cell dysfunction/exhaustion, stromal remodeling, and enhanced invasive behavior [78]. More broadly, cervical tumors can also recruit or induce suppressive myeloid compartments, including regulatory dendritic cells, M2-like macrophage programs, and MDSC-like populations, that dampen cytotoxic T-cell responses via immunosuppressive cytokines such as IL-10, TGF-β, metabolic suppression via the arginase/ROS pathways, for instance, and impaired priming, collectively reinforcing a permissive microenvironment for progression [79,80].

#### 3.4.3. Immune Exclusion vs. Immune Inflamed Phenotypes

Tumors are commonly classified into three major immunophenotypes based on the localization of cytotoxic immune cells: immune-inflamed, immune-excluded, and immune-desert phenotypes [81]. Immune-inflamed (“hot”) tumors are characterized by dense infiltration of CD8^+^ T cells within tumor nests, active interferon-γ signaling, frequent PD-L1 expression, and a pre-existing antitumor immune response, features that generally predict responsiveness to immunotherapy [82]. In contrast, immune-excluded tumors—considered a form of “cold” tumors—exhibit substantial immune cell accumulation at the tumor periphery or stroma, but limited penetration into the tumor parenchyma due to stromal, vascular, or chemokine-mediated barriers [83,84]. Emerging evidence suggests that cervical cancer, particularly in early or in situ stages, may preferentially display an immune-excluded phenotype, wherein T cells are present but functionally ineffective because they fail to infiltrate tumor tissue. This may account for the observed positive correlation between T-cell abundance and disease progression, as immune cells remain spatially and functionally disconnected from malignant epithelial cells [85].

At a broader level, immune phenotypes reflect distinct underlying biological mechanisms. Immune-desert tumors arise from immunologic ignorance or defective T-cell priming, immune-excluded tumors are shaped by stromal and vascular constraints, and immune-inflamed tumors may still evade immune destruction through T-cell exhaustion, immunosuppressive cell populations, or tumor-intrinsic inhibitory pathways such as MHC class I downregulation [81]. Understanding where cervical cancer lies along this immune continuum has important implications for immunotherapeutic strategies, as immune-excluded tumors may require combination approaches targeting stromal barriers or immune trafficking rather than checkpoint inhibition alone.

#### 3.4.4. Histologic and Molecular Subtypes Correlate with Differences in the Tumor Microenvironment

Taken together, these immune, stromal, and myeloid programs suggest that differences in HPV association and histologic subtype are accompanied by distinct tumor microenvironmental states, which may partially explain the heterogeneity in immune infiltration, immune exclusion, and therapeutic responsiveness observed across cervical cancer subtypes.

#### 3.4.5. Mechanistic Integration of HPV Oncogenic Signaling, Stromal Remodeling, and Immune Suppression

Collectively, the pathways described above converge on a coordinated HPV-driven program in which tumor-intrinsic oncogenic signaling and microenvironmental reprogramming are mutually reinforcing rather than independent processes. HPV-associated activation of telomerase, PI3K/AKT/mTOR, and Wnt/β-catenin signaling creates a permissive intracellular state that facilitates sustained paracrine communication with stromal and immune compartments [52,56,61]. These oncogenic programs interface with TGF-β and Wnt-mediated fibroblast activation, promoting extracellular matrix remodeling and chemokine gradients that physically and functionally restrict immune cell access to tumor epithelium [65,75]. In parallel, HPV oncoprotein–mediated attenuation of antigen presentation and innate immune sensing lowers immune pressure at the epithelial level, while stromal and myeloid signaling circuits amplify this effect by biasing immune trafficking and polarization toward suppressive and non-cytotoxic states [69,70,73,79]. The net result is not immune absence, rather immune mislocalization and dysfunction (immune exclusion or exhaustion), whose prevalence varies by molecular and histologic subtype [81,84,85]. This integrated framework explains how HPV oncogenesis simultaneously drives malignant progression, stromal restructuring, and immune evasion, thereby giving a plausible explanation for subtype differences and limited responses to immunotherapy.

### 3.5. Epigenetics

Epigenetic remodeling is another mechanism of regulation when it comes to cervical cancer biology. Tornesello et al. showed the clear dysregulation of non-coding RNAs, including microRNAs, long non-coding RNAs, and circular RNAs in cervical cancer, thus influencing tumorigenesis, invasion, metastasis, and resistance to chemo-radiotherapy [86]. Specific miRNA signatures associated with high-risk HPV infection vary across disease stages and show promise as diagnostic and prognostic biomarkers, including circulating miRNAs that may support non-invasive early detection and recurrence monitoring [86,87]. Long non-coding RNAs such as HOTAIR, MALAT1, and MEG3 are detectable in serum and correlate with metastatic phenotype, histologic subtype, lymph node spread, and recurrence risk [88,89]. Circular RNAs are increasingly recognized as additional regulators that function through miRNA sponging. In fact, the way they are differently expressed between malignant and normal tissue suggests emerging diagnostic and therapeutic potential [90]. Epigenetic silencing of Wnt antagonists such as SFRPs and Klotho supports EMT and invasion by reinforcing Wnt/β-catenin activation, illustrating convergence between epigenetic and signaling drivers [91]. Overall, these epigenetic programs align with HPV oncoproteins and genomic changes to shape tumor phenotype and progression.

### 3.6. Link to Clinical Treatments

Together, the HPV-driven disruption of p53 and pRb, telomerase upregulation, activation of PI3K/AKT/mTOR and Wnt/β-catenin signaling, genomic subtype heterogeneity, immune escape, and epigenetic remodeling support the development of more precise therapeutic approaches for cervical cancer. E6 and E7 are constitutively expressed tumor antigens that drive malignancy and survival, making them attractive therapeutic targets for vaccines and adoptive cellular therapies [92,93]. Genomic findings identifying frequent PI3K–MAPK and TGF-β pathway alterations support targeted approaches, including PI3K or mTOR inhibition [62]. The immune landscape, particularly PD-L1 upregulation and antigen-presentation disruption, provides a strong rationale for checkpoint blockade and immune-modulating combinations [94,95]. Finally, epigenetic signatures involving miRNAs, lncRNAs, and circRNAs represent promising biomarker and therapeutic avenues that may eventually enable more personalized treatment tailoring [86]. These interlocking molecular signatures therefore bridge pathophysiology to the expanding clinical toolkit in cervical cancer.

## 4. Targeted Therapy

### 4.1. Anti-Angiogenic Agents

**Figure 1:**
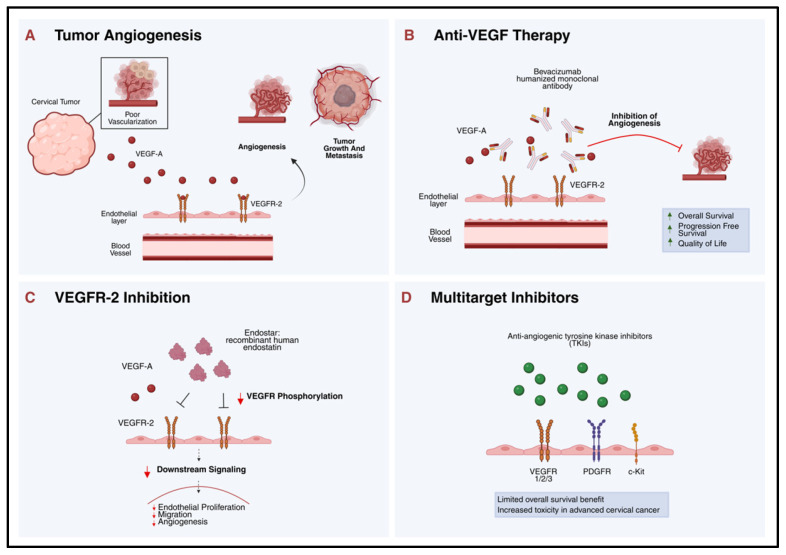
Anti-angiogenic therapies in cervical cancers. (**A**) The process involves the production of vascular endothelial growth factor A (VEGF-A) that binds VEGF receptor-2 (VEGFR-2) expressed by the endothelial cells, causing neovascularization that favors the development of tumors. (**B**) Bevacizumab, a humanized monoclonal antibody, inhibits VEGF-A by preventing it from binding to VEGF receptors, thus inhibiting angiogenesis and improving overall survival and progression-free survival in recurrent or metastatic cervical cancer without worsening the quality of life. (**C**) Endostar, a recombinant human endostatin protein, inhibits angiogenesis by downregulating VEGFR-2 expression and inhibiting VEGF signaling. (**D**) The anti-angiogenic tyrosine kinase inhibitors (TKIs) block several angiogenic receptors, such as VEGFR 1, 2, and 3, as well as Platelet-Derived Growth Factor Receptor (PDGFR) and c-Kit, but have been associated with minimal improvement in overall survival and increased toxicity in advanced cervical cancers.

The concept of tumor-induced angiogenesis was first coined by Judah Folkman in 1971, stating that tumors secrete pro-angiogenic factors for neovascularization to enhance tumor growth [96]. Since then, several anti-angiogenic agents have been developed, with bevacizumab earning FDA approval in 2004 for the treatment of metastatic colorectal cancer. Bevacizumab, a humanized monoclonal antibody, targets the Vascular Endothelial Growth Factor A (VEGF-A), preventing its interaction with the VEGF receptors (VEGFR) and thus inhibiting angiogenesis (See Figure 1) [97].

#### 4.1.1. Bevacizumab

From 2002 to 2006, Monk et al. [8] assessed the efficacy of bevacizumab alone in the treatment of persistent or recurrent squamous cell carcinoma (SCC) of the cervix in a phase II trial. While their sample size was small (46 participants) with no control group, their results suggested that bevacizumab had favorable outcomes (PFS: 3.4 months; OS: 7.29 months) when indirectly compared with other treatment regimens available at the time. Later, Tewari et al. [9,10] conducted an open-label phase III RCT, GOG-240, to further evaluate the efficacy of bevacizumab in combination with chemotherapy (cisplatin + paclitaxel or topotecan + paclitaxel) in women with persistent, recurrent, or metastatic cervical cancer. The second interim analysis showed superiority of the bevacizumab group as compared with the chemotherapy-only group (OS: 17 months vs. 13.3 months; HR: 0.71; *p*-value = 0.004). These results led to the FDA approval of bevacizumab for the treatment of women with advanced cervical cancer in 2014. Their final analysis confirmed the therapeutic benefit of bevacizumab addition (OS: 16.8 months vs. 13.3 months; HR: 0.77; *p*-value = 0.007). Importantly, patients in the bevacizumab arm did not have any significant quality of life deterioration compared with the chemotherapy group [98]. Common adverse events attributed to bevacizumab included grade 2 hypertension, thromboembolic events, neutropenia, and gastrointestinal (GI)/genitourinary (GU) fistula formation [9,10]. Compared to non-bevacizumab regimens, GI and GU fistula were 4.03 and 4.71 times more likely to occur in bevacizumab-treated patients [99]. To note, fistulae occurred exclusively in patients who had previous radiotherapy [10]. Two single-arm phase II trials later assessed the combination of bevacizumab with carboplatin + paclitaxel backbone chemotherapy, showing an overall response ranging from 79% to 88% [11,12]. One of these studies, JGOG1079, showed that patients receiving maintenance bevacizumab had a prolonged PFS compared with those who did not (14.3 months vs. 7.4 months; *p*-value = 0.0449) [12]. The CECILIA trial demonstrated rates of fistula formation with the bevacizumab, carboplatin, and paclitaxel combination similar to those reported in the GOG-240 trial [100]. In addition, a phase II trial (RTOG 0417) evaluated the safety and efficacy of chemoradiation with bevacizumab for stage IB-IIIB cervical cancer. It showed that the addition of bevacizumab is safe, with a 3-year OS of 81.3% [13,101]. To date, no phase III trial has further investigated the efficacy of the combination of chemoradiotherapy and bevacizumab for the treatment of locally advanced cervical cancer.

#### 4.1.2. Endostar

Alternatively, Endostar, a recombinant human endostatin, inhibits angiogenesis by interfering with the VEGF pathway and decreasing VEGFR-2 expression [102]. Two prospective studies evaluated the combination of Endostar and chemoradiation (CRT + E) in locally advanced cervical cancer [14,15]. Ke et al. [14] demonstrated significantly improved 1-year survival in the CRT + E group compared with the CRT arm (100% vs. 84.62%; *p*-value < 0.05), although the sample size was small (n = 52). In parallel, Lu et al. [15] found no significant difference in PFS between the two arms (HR: 0.496; *p*-value = 0.091) in a larger sample of 116 participants. However, in subgroup analysis stratified by VEGFR-2 tumor status, the CRT + E arm had an improved PFS compared with CRT alone (HR: 0.385; *p*-value = 0.026), suggesting that VEGFR-2 positive patients could benefit from Endostar addition to their treatment regimen [15]. In addition, a meta-analysis conducted by Maimaitiming et al. [103] examined 13 studies (7 RCTs, 4 NRCTs, and 2 cohorts) on CRT vs. CRT + E. The RCTs were of moderate quality, with high risk for performance and detection bias. Based on RCTs, CRT + E has a significantly 4.07 and 3.44 times higher chance of achieving ORR and DCR, respectively, compared to CRT alone [103].

#### 4.1.3. Tyrosine Kinase Inhibitors (TKI)

Furthermore, several other anti-angiogenic agents have been studied for the treatment of advanced and recurrent cervical cancer. First, Pazopanib, an oral TKI targeting VEGFR, PDGFR, and c-kit, and Lapatinib, an oral TKI targeting EGFR and Her2/neu, were evaluated alone and in combination in a phase II open-label randomized trial [104]. Participants in this trial were women with stage IVB recurrent/persistent cervical cancer who had received at least one therapeutic regimen for metastasis. 9% of patients on Pazopanib and 5% of those on Lapatinib achieved tumor response, and OS was increased by 11.6 weeks in the Pazopanib arm compared to Lapatinib (*p*-value = 0.045). However, these results need to be interpreted carefully, as patients discontinued treatment after a median duration of 11 and 13 weeks, respectively, for Pazopanib and Lapatinib, and more patients (61% vs. 55%) remained on a different antineoplastic regimen in the Pazopanib group [104]. Additionally, the combination arm was discontinued due to crossing the futility boundary with the Pazopanib arm and increased adverse events. In parallel, a phase II single-arm trial assessed the objective response rate in patients with metastatic or unresectable cervical cancer treated with sunitinib, an oral TKI targeting VEGF, c-Kit, and PDGF [105]. With a sample size of 19, no patient achieved a tumor response, and 26% developed a fistula during the study. Additionally, a phase II single-arm trial looked at the efficacy of Apatinib, a TKI targeting VEGFR-2, as second line in advanced or recurrent cervical cancer [106]. Out of the 20 enrolled participants, none achieved a complete tumor response, and only 3 (15%) achieved a partial tumor response [106]. On the other hand, a second phase II open-label RCT assessing Apatinib in combination with chemotherapy or CRT found no difference in OS (*p*-value = 0.72) but a significantly prolonged PFS in the Apatinib arm (HR: 0.44; *p*-value < 0.01) [107]. However, this study had a high risk of bias (funded by the drug manufacturer and trial registration occurred after trial completion), and the results need to be interpreted carefully. Finally, Symonds et al. [108] evaluated the efficacy of Cediranib, a TKI targeting VEGFR-1, 2, and 3, in combination with carboplatin and paclitaxel in patients with recurrent or metastatic cervical cancer in a phase II double-blind RCT [108]. They found no significant difference in OS (*p*-value = 0.42) but a significantly prolonged PFS (HR: 0.58, *p*-value = 0.032) [108].

#### 4.1.4. Vascular Normalization

While the reasons behind the limited efficacy of TKIs are unclear, a novel anti-angiogenesis mechanistic framework could provide some hints. Even though tumors induce angiogenesis, the newly formed vessels are different from their normal counterparts. Indeed, these vessels are more tortuous, heterogeneous, and leaky, which leads to increased interstitial pressure and decreased chemotherapy diffusion into the tumor milieu. In addition, this abnormal vascular architecture causes increased vascular resistance, ultimately leading to hypoxia and selection of aggressive phenotypes. Finally, the fenestrated nature of these vessels and the lack of pericytes ease metastasis mechanisms. While it was originally thought that one should starve tumor cells, the concept of vascular normalization (VN)—i.e., restoring normal tumor vascular architecture—is gaining ground, even though paradoxical. For instance, bevacizumab was shown to decrease tumor interstitial fluid pressure (IFP) and increase pericytes covering the vasculature in breast cancer patients [109]. Furthermore, VN relies on a balance of angiogenic signals, with low doses of bevacizumab enhancing this phenomenon while high inhibition lead to rapid vessel regression rather than repair [110]. VS enhances chemotherapy delivery by diffusion through reducing IFP and could decrease metastasis by increasing pericytes coverage and decreasing leakiness [111]. Pericytes, which reduce endothelial cells proliferation, migration and leakage, are PDGFR signaling for attachment and survival [112]. Hence, the decreased efficacy of TKIs could be attributed to PDGF signaling inhibition and their broad targeting, impeding vascular normalization [109,110,112].

### 4.2. Antibody-Drug Conjugates

**Figure 2:**
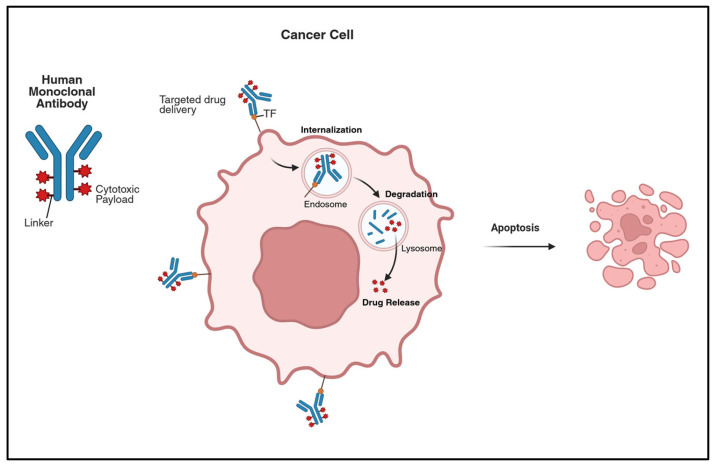
Antibody–Drug Conjugates (ADCs) as targeted therapy in cervical cancer. The three main components of an ADC are a highly potent drug payload, a cleavable linker that is relatively stable in the bloodstream, and a monoclonal antibody that is specific to tumor-associated antigens in the target cell membrane. The mechanism of ADCs is as follows: the monoclonal antibody binds to the antigen overexpressed on the surface of the target cell membrane; then it is endocytosed into the cell; the linker is cleaved; the potent anticancer drug is released in the cell, killing the target cells without affecting the host body. Tisotumab vedotin is an example of ADC, used in the treatment of cervical cancer; it targets tissue factor (TF) and has a component of Monomethyl auristatin E (MMAE) in microtubule polymerization inhibitors. ADCs make it possible to specifically target highly potent drugs to tumor cells, thus improving anti-tumor effects and minimizing off-target toxicity.

Antibody-Drug Conjugates (ADCs) represent a relatively new weapon in the armamentarium of anti-neoplastic agents. They are formed by three main constituents: a monoclonal antibody (mAb), a linker, and a cytotoxic payload. The mAb component permits a precise recognition of tumor-specific antigens, leading to increased efficacy and decreased systemic toxicities. The linker is designed to allow for a stable conjugation between the mAb and the drug in the blood, yet it can be degraded once inside the targeted cells. This new technology has opened the door for the use of drugs that have detrimental adverse effects when given systemically and blindly (See Figure 2) [113].

Tisotumab Vedotin (TV) is an ADC with a human mAb targeting tissue factor (TF), conjugated to monomethyl auristatin E (MMAE), an inhibitor of tubulin polymerization [16]. Compared to the adjacent normal cells, TF is upregulated in cervical cancer and plays a role in tumor invasion and metastasis [114]. The innovaTV 201 phase I/II study assessed the safety and efficacy of TV in patients with recurrent or metastatic cervical cancer who progressed on a platinum-based regimen. In this trial, 55 patients were enrolled, with 91% having previously received taxanes and 67% bevacizumab in combination with chemotherapy. The ORR was 24%, with no patient achieving a complete response [16]. In parallel, the innovaTV 204 phase 2 open-label trial demonstrated an ORR of 24% in 101 patients with recurrent or metastatic cervical cancer having received doublet chemotherapy plus bevacizumab, with 7 patients reaching a complete response. Common adverse events included alopecia, epistaxis, nausea, conjunctivitis, dry eyes, and fatigue [17]. This trial led to the accelerated FDA approval of TV for metastatic or recurrent cervical cancer in September 2021. Later, the innovaTV 301 phase 3 open-label RCT evaluated the efficacy of TV in patients with metastatic or recurrent cervical cancer who progressed on or after standard chemotherapy plus bevacizumab and anti-PD1/anti-PD-L1 [18]. This study revealed significantly prolonged OS (11.5 months vs. 9.5 months; HR: 0.70; *p*-value = 0.004) and PFS (4.2 months vs. 2.9 months; HR: 0.67; *p*-value < 0.001). While 14.8% of patients in the TV arm discontinued treatment due to adverse events, compared to 3.8% in the chemotherapy group, 13.9% of patients reported an improved quality of life at cycle 5 with TV, compared to 3.4% with chemotherapy [18]. The results of this trial were the basis for the traditional FDA approval of TV for recurrent or metastatic cervical cancer patients who progressed during or after chemotherapy in April 2024. Finally, Vergote et al. [19] assessed the safety and efficacy of TV in combination with carboplatin (arm D), pembrolizumab (anti-PD1) as first line (arm E) or second/third line (arm F) in a phase 1b/2 single-arm trial. They found promising responses in the different arms, with ORRs of 54.5%, 40.6%, and 35.3%, respectively, in arms D, E, and F [19]. Even though the sample size was small, these results open the field for further trials to explore the efficacy and safety of these combinations. Importantly, all trials showed that treatment response was independent of tumor TF expression [17,18,19].

In parallel, Bulumtatug Fuvedotin (BFv) is an ADC targeting Nectin-4 and conjugated to MMAE that has recently been studied in cervical cancer. Indeed, a phase I/II open-label trial evaluated the efficacy and safety of BFv in 62 patients with advanced cervical cancer who failed on one or more lines of systemic treatment [20]. The study reported an ORR of 32.1% in these patients, with subgroup analysis showing a 50% ORR among patients with high Nectin-4 expression [20].

Furthermore, there is an increased interest in the interplay between ADCs and the immune TME. This bidirectional relationship has been studied in different cancers, with lessons learned that could shape future therapeutic developments. First, tumor-associated macrophages (TAMs), which classically contribute to cancer cells’ survival through immune modulation, play a role in bystander killing of tumor cells by ADCs. Indeed, TAMs can internalize ADCs through Fc receptor interactions and release the payloads into the TME [115]. In fact, TAMs’ immune depletion resulted in lower efficacy of ADCs [116]. However, the concentration of payload in nearby cells through the bystander killing effect is lower than through direct ADCs binding to tumor cells. In addition, the immunologic tumor cell death leads to the release of immunogenic signals such as damage-associated molecular patterns (DAMPs) and an increase in pro-inflammatory cytokines, transforming the TME into an immune-inflamed milieu by promoting phagocytosis and dendritic cells activation and maturation [117].

## 5. Immunotherapy

**Figure 3:**
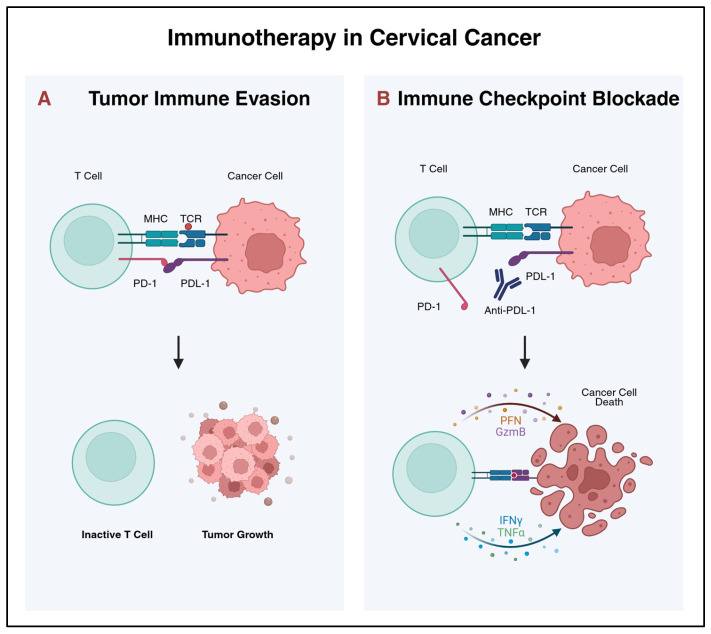
Immune checkpoint blockade restores T cell cytotoxicity through PD-1/PD-L1 inhibition in cervical cancer. (**A**) The interaction of the tumor cell surface peptide MHC and the T cell receptor from a cytotoxic T cell recognizes the tumor antigens. Tumors overexpress the protein programmed death-ligand 1 (PD-L1) to evade the destruction of the immune cells. T cell exhaustion is caused by the PD-L1/PD-1 interaction in the T cells activated against the tumor. It triggers the immune inhibitory signaling pathway, thus reducing T cell proliferation, cytokine secretion, and cytotoxic effects. (**B**) The immune checkpoint inhibitors block the PD-1/PD-L1 interaction. The cytotoxic T cells are no longer inhibited by tumor cells and can mediate cell death by releasing granzymes and perforins.

Cervical cancer is an ideal candidate for immunotherapy. Often associated with HPV infection, cervical cancer cells express oncoproteins E6 and E7, which make them recognizable by the immune system [118]. However, as outlined above, cervical tumors evolve within an immunosuppressive microenvironment [69]. With its potential to overcome these immune-evasion strategies, immunotherapy appears to be a promising treatment for both advanced and locally advanced settings (See Figure 3) [1].

Although most cervical cancers are HPV-positive, only a subset of patients benefits from immunotherapy. In trials of PD-1 inhibitors, objective response rates have been modest [21]. This discordance is due to the disruption of antigen presentation and immune recognition by HPV oncoproteins through mechanisms that go beyond increased PD-L1 expression. MHC class I reduced expression through HPV’s E5 oncoprotein is a cause of primary resistance to immunotherapy, as described previously. One study found that, despite the fact that 84% of their cervical squamous cell carcinoma samples were PD-L1 positive, more than one-third (35.5%) showed clonal or complete loss of MHC class I expression. This indicates an impaired antigen presentation despite checkpoint ligand expression [119]. One common adaptive resistance mechanism is the compensatory rise in alternate inhibitory receptors after PD-1 is blocked. Following this blockage, tumors are prone to increased expression of the inhibitory immune checkpoint receptors CTLA-4, TIGIT, LAG-3, and TIM-3 [120]. CheckMate 358 showed that combined PD-1 + CTLA-4 inhibition in advanced cervical cancer can induce deeper and more durable responses in some patients who might not respond to anti-PD-1 alone [22]. In addition, cervical cancer TME ranges from immune-hot to immune-cold, profoundly influencing immunotherapy outcomes. Some immune cold tumors lack the active immune cells needed for PD-1/PD-L1 inhibitors to act [121]. Moreover, the cellular composition of the TME can modulate the effectiveness of therapy: abundant Tregs and M2 tumor-associated macrophages (TAMs) secrete IL-10, TGF-β, and other factors that suppress effector T-cell function. Exhausted CD8^+^ tumor-infiltrating lymphocytes in cervical cancer frequently express alternative inhibitory receptors such as CD96. Recent single-cell analyses have shown that PD-1^+^CD96^+^CD8^+^ T cells represent a terminally exhausted population characterized by diminished cytokine production, poor cytotoxic function, and is associated with inferior survival. Importantly, CD96 expression is further upregulated following PD-1 blockade, suggesting an adaptive resistance mechanism [122].

Furthermore, Bao et al. used single-sample gene set enrichment analysis (ssGSEA) to find that a higher risk score of five immune-related prognostic biomarkers (HLA-DMA, DMBT1, CXCR6, CX3CL1, and SEMA3A) was associated with worse prognosis, possibly through regulating chemokine-mediated signaling and receptor-ligand interactions [123]. Being directly related to immunotherapy targets, these results offer new insights to predicting treatment responses.

KEYNOTE-158 is a phase II trial that demonstrated the efficacy and safety of pembrolizumab monotherapy in PD-L1-positive patients, being non-randomized and single-armed [23]. The result of this trial led to the first approval of pembrolizumab by the FDA in 2018 as a second-line treatment for patients with recurrent or metastatic cervical cancer who progressed on or after chemotherapy and have a CPS ≥ 1. Subsequently, in 2021, Pembrolizumab received the FDA approval to be used in combination with platinum-based chemotherapy (with or without bevacizumab) as a first-line regimen for patients with persistent, recurrent, or PD-L1–positive tumors (CPS ≥ 1) metastatic disease. This approval was granted based on the results of KEYNOTE-826, a randomized, double-blind, phase III trial including a globally diverse population evaluating pembrolizumab (200 mg) or placebo plus platinum-based chemotherapy and bevacizumab according to investigator choice. The combination of chemotherapy with pembrolizumab showed improved survival, with mPFS of 10.4 vs. 8.2 months (HR 0.62) and 24-month OS of 53.0% vs. 41.7% (HR 0.64) compared to chemotherapy alone [21].

In parallel, even though ipilimumab, a CTLA-4 inhibitor, showed minimal efficacy in cervical cancer on its own [24], its use in combination with anti–PD-1 antibodies has revealed potential clinical benefit. The phase I/II open-label randomized multicohort study CheckMate 358 evaluated nivolumab immunotherapy for treating recurrent or metastatic cervical cancer through two different dosing schedules of nivolumab with ipilimumab added as combination therapy. The research demonstrated that combination therapy achieved better objective response rates (ORRs) ranging from 31% to 40% than nivolumab monotherapy, which obtained an ORR of about 26% [22]. In patients with recurrent cervical cancer after first-line platinum-containing chemotherapy, the EMPOWER-Cervical 1 (GOG-3016/ENGOT-cx9) trial demonstrated that cemiplimab improved overall survival compared with chemotherapy. At a median follow-up of 47.3 months (data cut-off: 20 April 2023), median OS was 11.7 months in the cemiplimab group versus 8.5 months in the chemotherapy group [25].

Combination therapy might help escape some of these resistance mechanisms, but these approaches might come at a cost of increased toxicity. Immunotherapy must also be balanced against its potential toxicities. In KEYNOTE-826, grade ≥ 3 treatment-related adverse events were observed in 81.8% of the immunotherapy plus chemotherapy arm vs. 75.1% of the chemotherapy alone arm. The rate of immune-related adverse events doubles when combining pembrolizumab with chemotherapy (33.9% vs. 15.2% with chemotherapy alone) [21]. Additionally, immunotherapy combinations do carry greater immune toxicity. In CheckMate 358, 42% of patients receiving the nivolumab + ipilimumab experienced serious adverse events vs. 16% with nivolumab alone, including one treatment-related death due to immune-mediated [22]. These immune-related toxicities represent the trade-off for improved efficacy when trying to overcome the multifactorial mechanisms behind cervical cancer resistance.

As for locally advanced cervical cancer (LACC), concurrent chemoradiotherapy (CCRT) is the standard treatment [3]. Many studies have tried to evaluate the benefit of adding immunotherapy with CCRT in LACC. CALLA was a randomized phase III trial comparing durvalumab plus CCRT with placebo plus CCRT in patients with LACC, but did not demonstrate a significant improvement in progression-free survival [26]. In contrast, the KEYNOTE-A18 trial showed improved 24-month progression-free survival with pembrolizumab plus CCRT compared with placebo (68% vs. 57%; HR for progression or death, 0.70; 95% CI, 0.55–0.89), although no significant overall survival benefit was observed at 24 months (87% vs. 81%; HR for death, 0.73; 95% CI, 0.49–1.07) [27].

## 6. Therapeutic Vaccines

**Figure 4:**
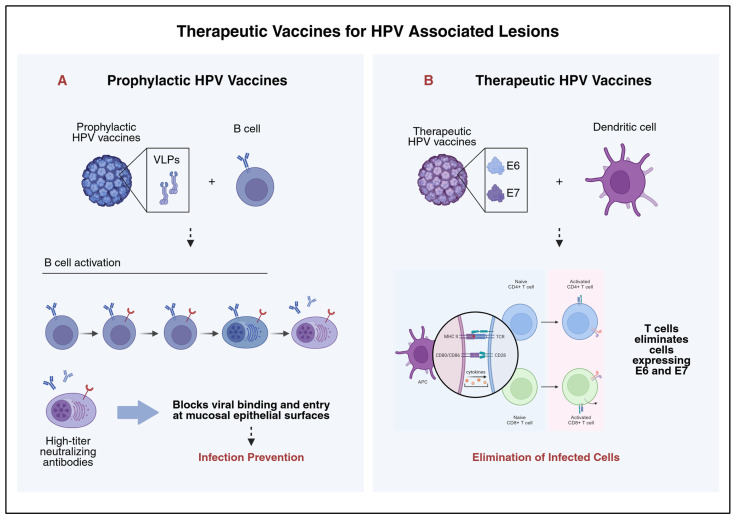
Prophylactic and Therapeutic HPV Vaccines. (**A**) HPV prophylactic vaccines are made up of virus-like particles (VLPs), whose structure closely resembles the viral capsid proteins, namely L1, of the human papillomavirus. The virus-like particles are noninfectious, highly immunogenic, and capable of inducing the activation of B cells accompanied by affinity maturation. High levels of neutralizing antibodies are generated, offering protection from human papillomavirus infection by inhibiting the viral attachment and entry into the epithelial cells in the mucosal surfaces. (**B**) Conversely, therapeutic HPV vaccines induce immune activation targeted at the cell-mediated immune response. These vaccines comprise the oncogenic proteins E6 and E7 of HPV. These proteins are constitutively expressed in cancerous cells. Dendritic cell antigen presentation stimulates the activation of CD4+ helper T cells and the activation of CD8+ cytotoxic T lymphocytes. These cytotoxic lymphocytes selectively target E6/E7-expressing cells, focusing on HPV-dysplastic or HPV-infected cells, as opposed to protecting against primary infection.

Prophylactic HPV vaccination is the most effective long-term strategy for preventing high-risk HPV infections and the development of HPV-related cancers. Current vaccines are based on non-infectious virus-like particles (VLPs). These VLPs are composed of major capsid protein L1, which mimics the geometry of native virions but does not contain viral DNA [124]. Three VLP-based vaccines have been licensed worldwide: the bivalent Cervarix targeting HPV16 and HPV18 [125,126], the quadrivalent Gardasil, which adds coverage to HPV6 and HPV11 [127,128], and the nonvalent Gardasil9 that extends protection against HPV types 31, 33, 45, 52, and 58 [129,130]. All three vaccines have demonstrated high efficacy and an excellent safety profile. They provide near-complete protection when administered before HPV exposure [131]. These vaccines have been widely adopted in national immunization programs targeting adolescents. It is believed that their mechanism of action depends mostly on the production of strong type-specific and cross-neutralizing antibody responses, with serum antibody levels that are much higher than those that occur naturally (See Figure 4) [127,132,133].

Although prophylactic HPV vaccines provide effective protection against HPV16 and HPV18, which are the genotypes responsible for most cervical cancers, their impact is limited to preventing new infections rather than treating them. This means they have no therapeutic effect on individuals who are already infected [134]. Because of this, HPV-associated CIN remains common, and current management relies on ablative surgical procedures that can lead to long-term reproductive morbidity [28]. There is a substantial unmet need for immune-based therapeutic approaches that are capable of treating existing HPV- induced disease, especially CIN2/3, as many women remain unvaccinated and HPV infections continue to progress to high-grade lesions [134]. While preventive vaccines work on humoral immunity, therapeutic vaccines target cell-mediated immunity. Indeed, these new vaccines deliver HPV-specific antigens to antigen-presenting cells, which in turn prime and activate CD4+ and CD8+ T cells against cells expressing these antigens (See Figure 4) [135].

VGX-3100 is a vaccine with two plasmid DNAs encoding HPV16/18 E6 and E7 proteins. Its efficacy and safety in women with HPV16/18 positive CIN2/3 were evaluated in a randomized, double-blind, placebo-controlled phase 2b trial conducted by Trimble et al. VGX-3100 significantly increased histopathological regression compared to placebo (49.5% vs. 30.6%; *p*-value = 0.034). The vaccine also achieved significantly higher rates of concomitant regression and HPV16/18 viral clearance occurring in 40.2% of the vaccine recipients compared to 14.3% in the placebo group (*p*-value = 0.034). VGX-3100 elicited robust peripheral and tissue immune responses as well, with stronger CD8+ T-cell and antibody activity (*p*-value = 0.001) and higher combined E6/E7-specific T-cell responses (*p* < 0.0001), which is correlated closely with clinical regression [28]. Notably, responders showed a significantly higher frequency of perforin-producing T cells compared to non-responders, suggesting that the functionality of T cells—rather than just their presence—is a key biomarker for stratification. In parallel, GX-188E is composed of a plasmid DNA encoding HPV 16/18 E6 and E7 antigens, linked to Flt3L and tpa to enhance activation of dendritic cells. A multicenter phase II clinical trial done by Choi et al. [29] evaluated GX-188E in women with HPV16/18-positive CIN3. The vaccine demonstrated notable clinical efficacy and immunologic activity, demonstrating histopathological regression in 52% of participants at 20 weeks and 67% by 36 weeks. Regression was strongly associated with concurrent HPV clearance (20 weeks: OR = 13.867, *p* < 0.001; 36 weeks: OR = 25.313, *p*-value < 0.001). This trial identified HPV genomic variants as a major predictor of success, as specific variations in the HPV16 E6 and E7 regions were significantly associated with whether a patient achieved tumor regression. Additionally, lesion size served as a practical clinical biomarker; patients whose lesions covered less than 50% of the cervix had significantly higher regression rates. However, these findings need to be validated in larger trials. Women who achieved regression also exhibited significantly greater HPV-specific IFN-γ ELISpot responses compared with non-regressors (*p*-value = 0.028). This indicated that the therapeutic effect of GX-188E is tightly linked to the induction of robust antigen-specific cellular immunity [29].

In addition, Rosales et al. looked at the therapeutic efficacy of the MVA E2 vaccine (a recombinant virus with the bovine papilloma virus E2 protein) in a phase III single-arm trial in Mexico. This study included both women with CIN1, 2, or 3 and men with urethral condyloma or anal lesions and showed histological regression in 89% of women and 100% of men. However, only 73% of women with high-grade lesions achieved regression. This trial is limited by the absence of a control group, as it is not possible to determine the rate of clearance caused by natural regression [30].

Furthermore, Kawana et al. conducted a randomized phase I/II trial on the oral HPV-16 E7 therapeutic vaccine (IGMKK16E7). The vaccine produced significantly higher complete regression rates (31.7%) in women with CIN2/3 compared with placebo (12.5%) at 24 weeks (rate difference 95% CI [0.5, 37.8]). The strongest effects were shown in HPV16 only (*p*-value = 0.026) and CIN3 subgroups (*p*-value = 0.011). The vaccine was safe and well-tolerated. It also induced a dose-dependent increase in HPV-16 E7-specific IFN-γ-producing T-cells, which supports a true immunologic mechanism of lesion regression [31].

Most recently, Eerkens et al. evaluated the therapeutic vaccine Vvax001 in women with HPV16-positive CIN3. The vaccine achieved histopathological regression in 50% (9/18) of patients. It also elicited lesion-size reduction in 94% (17/18) of patients, with HPV16 clearance observed in all evaluable regressors and 25% of non-regressors. However, it did not elicit clearance of other HPV types [32]. Despite a small sample size (n = 18) and signle arm design, the study provided critical insights into the tumor microenvironment (TME). It identified that a pre-existing “immune-inflamed” state is crucial for vaccine success. In contrast, patients with an “immune-excluded” TME were less likely to respond.

## 7. Ongoing Trials

To build on the advances that have been made in molecularly targeted and immune-based therapies, several ongoing clinical trials are further investigating novel agents and combination approaches for cervical cancer to evaluate their safety and efficacy. A particularly active area of investigation is the integration of immunotherapy into earlier stages of cervical cancer, alongside continued efforts to overcome resistance in recurrent or metastatic settings, through HPV-targeted adoptive cellular therapy, vaccine-based therapy, and a bispecific antibody approach to overcome checkpoint resistance.

Multiple ongoing clinical trials are currently investigating neoadjuvant chemo-immunotherapy approaches that incorporate immunotherapy at early stages to improve tumor responses to chemotherapy, before surgery. Particularly, a phase II trial (NCT07003620) is investigating the combination of paclitaxel-carboplatin with PD-1/PD-L1 inhibition in patients with locally advanced cervical cancer (FIGO IB3–IIB, tumor size > 4 cm). Patients will undergo radical hysterectomy with pelvic lymphadenectomy after three cycles of treatment, and tumor samples will subsequently be analyzed using single-cell RNA sequencing to explore mechanisms underlying differential treatment responses. This study represents a relatively conservative approach to immune integration, in which a single-axis checkpoint is added to standard chemotherapy. It also aims to correlate the patients tumor’s molecular characteristics with treatment response, an important step towards precision medicine.

In parallel, the open-label non-randomized phase II trial (NCT06878222) is currently evaluating dual-checkpoint blockade as upfront immune intensification for locally advanced cervical cancer (FIGO IB3, IIA2, IIB, or IIICr). This study combines Iparomlimab and Tuvonralimab, targeting the PD-1 and CTLA-4 pathways, along with Paclitaxel and Cisplatin as neoadjuvant therapy prior to surgery if CR or PR is achieved. Primary outcomes include ORR and pathological complete response, alongside analysis of tumor-related biomarkers. This study could provide an additional therapeutic option, especially for patients who do not respond to or develop resistance to single-line immunotherapy, while balancing the safety profile.

On the other hand, an ongoing phase I study (NCT05357027) is evaluating the safety and tolerability of TC-E202, a genetically engineered T cell targeting HPV E6 protein, in HPV16-positive patients. Patients’ T cells are first isolated and then genetically engineered to express a T-cell receptor specific for the HPV E6 antigen, generating cells that demonstrate reactivity against HPV16-positive tumor targets. This study specifically aims to determine the recommended phase II dose, using a standard 3 + 3 dose-escalation design in patients with recurrent or metastatic HPV16-positive cervical carcinoma. Patients undergo a non-myeloablative lymphodepleting regimen consisting of cyclophosphamide and fludarabine, followed by cell infusion and subsequent administration of interleukin-2 (IL-2). This trial builds on successful stories of engineered T cell therapy in hematological malignancies to further foster personalized medicine.

In addition, a phase II, open-label trial (NCT04580771) is evaluating the safety and toxicity profile of the immune nanoparticle liposomal HPV-16 E/E7 multi-peptide vaccine PDS0101 in combination with standard chemoradiation in patients with cervical cancer (Stage IB3-IVA). The primary outcome is the rate of grade ≥ 3 acute toxicity, with secondary outcomes looking at clinical efficacy. PDS0101 consists of two active components: R-DOTAP, which enhances immune activation against HPV viral proteins, and small HPV-derived peptide antigens. The clinical value of this approach depends on whether immune priming can translate into improved long-term control of cervical cancer without increasing the treatment dose and limiting toxicity in return.

Furthermore, targeted antibody monotherapy-based strategies have demonstrated limited efficacy, with reported response rates of only 10–20% in patients with recurrent or metastatic cancer. To address this limited activity, an ongoing phase II trial (NCT07141186) is evaluating the safety and efficacy of the bispecific antibody QL1706, which simultaneously targets PD-1 and CTLA-4 in this patient population. Eligible patients include those who have failed prior PD-1 inhibitor therapy. In line with the previously mentioned trial (NCT06878222), this study also aims to provide further lines of treatment once patients develop resistance to current first and second-line therapies with immunotherapy.

Finally, novel immune-modulatory combination strategies are being explored in recurrent or metastatic cancer. A phase I/II trial (NCT04287868) is currently exploring a multi-agent regimen combining PD50101 (therapeutic vaccine), M7824 (bifunctional fusion protein targeting PD-L1 and TGF-β), and NHS-IL12 (tumor-targeted immunocytokine) in patients with HPV-associated malignancies, including advanced cervical cancer. This study aims to assess the safety, tolerability, and preliminary efficacy of this multi-modal immune treatment regimen. The primary endpoint is ORR, while secondary outcomes include dose-limiting toxicities (DLTs), PFS, and treatment-related adverse events.

Collectively, these ongoing trials illustrate the expanding role of immunotherapy, vaccines, and targeted therapy in cervical cancer treatment, highlighting improvements in efficacy, safety, and clinical outcomes, while supporting integration of innovative approaches into standard treatment paradigms.

## 8. Translational Implications and Future Directions

Cervical cancer pathophysiology and available treatment resistance mechanisms offer guidance to future drug and combination therapy development. Indeed, ADCs’ penetration into the tumor milieu is often limited by the desmoplastic nature of the TME and increased IFP. Indeed, TGF-β signaling in cervical cancer increases the activity of stromal fibroblasts to deposit type I and III collagen, fibronectin and hyaluronan into the TME, forming a dense milieu that impedes ADCs penetration. Anti-TGF-β therapy has been studied in pre-clinical murine colorectal cancer models and was shown to increase the anti-tumor efficacy of ADCs [136], providing a mechanistic rationale for exploring similar strategies in cervical cancer. Similarly, low-dose bevacizumab was shown to induce vascular normalization in cervical cancer, decreasing interstitial pressure and facilitating drug diffusion. Lower drug levels could therefore achieve similar intra-tumor concentrations with decreased adverse events. Trials assessing combination therapies with bevacizumab are therefore warranted, to evaluate survival benefits, adverse events profile and overall quality of life.

In addition, modulating the immune TME could constitute a major therapeutic strategy in the future, enhancing the efficacy of immune checkpoint inhibitors. Future studies must move beyond the use of a single immunotherapy agent and instead focus on methods that transform immune-excluded tumors into immune-inflamed tumors. Neoadjuvant platinum-based chemotherapy may facilitate this shift by partially sensitizing tumors to immunotherapy [137]. This can be done through enhancing tumor antigen release, boosting antigen presentation, and promoting immune cell infiltration. However, in resistant tumors, this process is incomplete, necessitating additional measures. Targeted combinations, including STING agonists, may amplify innate immune activation and facilitate successful T-cell priming. Indeed, STING is a transmembrane protein that promotes a robust immune response by increasing the populations of CD8+ T cells and dendritic cells [138]. Additionally, ADCs promote an immune-inflamed milieu by releasing DAMPs and increasing dendritic cells’ maturation. It is thus reasonable to hypothesize that ADCs and immune checkpoint inhibitors may act synergistically. Even though this combination has not yet been investigated in cervical cancer, it has shown promising results in urothelial, breast, and gastric cancers [117]. Similarly, therapeutic vaccines were shown to have increased efficacy in an immune-inflamed milieu [32]. Therefore, similar combination therapies could be applied to synergistically combat cervical cancer cells, but further trials are needed to assess the safety profile and efficacy.

To date, response to immunotherapy is still heterogeneous, and biomarkers to guide treatment are needed. Even though the PD-L1 positive score is a clinically validated predictive biomarker, its specificity is still limited [23]. Other strong emerging candidates, such as MHC class 1 loss and distinction between T-cell–inflamed vs. immune-excluded phenotypes, may more effectively stratify patients and predict response to immunotherapy [119]. On the other hand, response to therapeutic vaccines depends on several factors, as showcased in previously mentioned trials. As such, lesion size, HPV type and immune TME could serve as future biomarkers for patients’ stratification.

## 9. Conclusions

Cervical cancer remains one of the most commonly diagnosed cancers in women, even though effective preventive vaccines are available worldwide. Fortunately, several lines of treatment targeting the molecular signatures of this cancer have been studied and are now being used for its treatment, especially in cases of recurrent, persistent, or metastatic disease. Targeting the tumor blood supply with VEGF inhibitors or directly delivering toxic payloads to the tumor with ADCs, in addition to modulating the tumor immune microenvironment with immunotherapy, are essential treatment modalities used clinically to halt this disease. Several therapeutic vaccines have been developed to enhance cell-mediated therapy, but more trials are needed before they can constitute an additional tool for the treatment of localized disease.

## Figures and Tables

**Table 1:** Summary of trials evaluating the safety and efficacy of targeted therapy, immunotherapy and therapeutic vaccines in cervical cancer.

*Study Title (Year)*	*Trial Design*	*Country*	*Population (Sample Size)*	*Intervention*	*Control*	*Primary Outcome*	*Reference*
Phase II Trial of Bevacizumab in the Treatment of Persistent or Recurrent Squamous Cell Carcinoma of the Cervix: A Gynecologic Oncology Group Study (2009)	Phase II	USA	Women with persistent or recurrent SCC of the cervix having received prior cytotoxic chemotherapy (n = 46)	Bevacizumab	NA	Median PFS: 3.4 months	[8]
Bevacizumab for advanced cervical cancer: final overall survival and adverse event analysis of a randomised, controlled, open-label, phase 3 trial (Gynecologic Oncology Group 240) (2015)	Phase III, open-label, randomized	USA, Canada, Spain	Women with metastatic, recurrent or persistent cervical cancer (n = 452)	Cisplatin + Paclitaxel + Bevacizumab (arm 1) OR Topotecan + Paclitaxel + Bevacizumab (arm 2)	Cisplatin + Paclitaxel OR Topotecan + Paclitaxel	OS: 16.8 vs. 13.3 months. HR 0.77, *p* = 0.0068 (Arm 1) OS: 15 vs. 17.5 months. HR 0.75, *p* = 0.04 (Arm 2) OS: 12 vs 16.2, HR 0.8, *p* = 0.15	[9,10]
Phase II trial of paclitaxel, carboplatin, and bevacizumab for advanced or recurrent cervical cancer (2019)	Phase II	Japan	Women with advanced or recurrent cervical cancer not amenable to curative treatment (n = 34)	Paclitaxel + Carboplatin + Bevacizumab	NA	ORR 88%	[11]
Paclitaxel-carboplatin and bevacizumab combination with maintenance bevacizumab therapy for metastatic, recurrent, and persistent uterine cervical cancer: An open-label multicenter phase II trial (JGOG1079) (2022)	Phase II, open-label, single-arm	Japan	Women with metastatic, recurrent, or persistent cervical cancer who did not receive prior chemotherapy (n = 69)	Paclitaxel + Carboplatin + Bevacizumab	NA	Median PFS: 11.3 months	[12]
RTOG 0417: Efficacy of Bevacizumab in Combination with Definitive Radiation Therapy and Cisplatin Chemotherapy in Untreated Patients with Locally Advanced Cervical Carcinoma (2014)	Phase II	USA, Canada, Saudi Arabia	Women with untreated locally advanced cervical cancer (n = 49)	Weekly Cisplatin + radiotherapy and 3 cycles of Bevacizumab	NA	3-year OS: 81.3%	[13]
Early Efficacy of Endostar Combined with Chemoradiotherapy for Advanced Cervical Cancers (2012)	Not mentioned	China	Women with advanced cervical cancer (n = 52)	Weekly Cisplatin + radiotherapy + Endostar	Weekly Cisplatin + radiotherapy	1-year OS: 100% vs. 84.62%, *p* < 0.05	[14]
Endostar, an Antiangiogenesis Inhibitor, Combined With Chemoradiotherapy for Locally Advanced Cervical Cancer (2021)	Phase II	China	Women with locally advanced cervical cancer (n = 116)	Weekly Cisplatin + radiotherapy + Endostar	Weekly Cisplatin + radiotherapy	2-year PFS: 80.8% vs. 63.5%, HR 0.496, *p* = 0.091	[15]
Tisotumab vedotin in previously treated recurrent or metastatic cervical cancer (2020)	Phase I/II, open-label	USA, UK, Belgium	Women with recurrent or metastatic cervical cancer who progressed on a platinum-based regimen (n = 55)	Tisotumab Vedotin	NA	ORR: 24%	[16]
Efficacy and safety of tisotumab vedotin in previously treated recurrent or metastatic cervical cancer (innovaTV 204/GOG-3023/ENGOT-cx6): a multicentre, open-label, single-arm, phase 2 study (2021)	Phase II, open-label single arm	USA, Europe	Women with recurrent or metastatic cervical cancer who progressed on or after doublet chemotherapy and bevacizumab (n = 101)	Tisotumab Vedotin	NA	ORR: 24%	[17]
Tisotumab Vedotin as Second- or Third-Line Therapy for Recurrent Cervical Cancer (innovaTV 301/ENGOT-cx12/GOG-3057) (2024)	Phase III, open-label randomized	Europe, North and Latin America, and the Asia Pacific	Women with recurrent or metastatic cervical cancer who progressed after first-line systemic therapy (n = 502)	Tisotumab Vedotin	Chemotherapy	Median OS: 11.5 vs. 9.5 months, HR 0.70, *p* = 0.004	[18]
Tisotumab vedotin in combination with carboplatin, pembrolizumab, or bevacizumab in recurrent or metastatic cervical cancer: results from the innovaTV 205/GOG-3024/ENGOT-cx8 study (2023)	Phase Ib/II, open-label	USA, Europe, Japan	Women with Recurrent or stage IVB cervical cancer (n = 142)	Arm D: Tisotumab Vedotin + Carboplatin Arm E: Tisotumab Vedotin + Pembrolizumab Arm F: 2nd/3rd line Tisotumab Vedotin + Pembrolizumab	NA	(Arm D) ORR: 54.5% (Arm E) ORR: 40.6% (Arm F) ORR: 35.3%	[19]
Bulumtatug Fuvedotin (BFv, 9MW2821), a next-generation Nectin-4 targeting antibody-drug conjugate, in patients with advanced solid tumors: a first-in-human, open-label, multicenter, phase I/II study (2025)	Phase I/II, open-label	China	Women with advanced cervical cancer (n = 62)	Bulumtatug Fuvedotin	NA	ORR: 32.1%	[20]
Pembrolizumab for Persistent, Recurrent, or Metastatic Cervical Cancer (2021)	Phase III, double-blind, randomised	Argentina, Australia, Canada, Chile, Colombia, Europe, Japan, Republic of Korea, Mexico, Peru, Russia, Taiwan, Turkey, United States	Women with persistent, recurrent, or metastatic cervical cancer (n = 617)	Pembrolizumab	Placebo + plus platinum-based chemotherapy + bevacizumab (per investigator discretion)	Median PFS: 10.4 months vs. 8.2 months (HR: 0.62)	[21]
Nivolumab with or without ipilimumab in patients with recurrent or metastatic cervical cancer (CheckMate 358): a phase 1–2, open-label, multicohort trial (2024)	Phase I/II, open-label	United States, United Kingdom, Europe, Republic of Korea, Mexico, Japan, Taiwan	Women with recurrent or metastatic cervical cancer (n = 193)	Nivolumab monotherapy, NIVO3 + IPI1, or NIVO1 + IPI3	NA	ORR 26% with nivolumab, 31% with NIVO3 plus IPI1, 40% with randomized NIVO1 plus IPI3, and 38% with pooled NIVO1 plus IPI3	[22]
Efficacy and Safety of Pembrolizumab in Previously Treated Advanced Cervical Cancer: Results From the Phase II KEYNOTE-158 Study (2019)	Phase II, open-label	Australia, Brazil, Canada, Europe, Japan, Russia, South Korea, Taiwan, United States	Women with multiple advanced solid tumor types that have progressed with standard-of-care systemic therapy (n = 98)	Pembrolizumab	NA	ORR 12.2% (95% CI, 6.5% to 20.4%), 3 complete and 9 partial responses.	[23]
Association of Ipilimumab With Safety and Antitumor Activity in Women With Metastatic or Recurrent Human Papillomavirus-Related Cervical Carcinoma (2017)	Phase I/II	Canada, United States	Women with cervical cancer (metastatic squamous cell carcinoma or adenocarcinoma) (n = 42)	Ipilimumab	NA	1 partial response and 10 stable disease	[24]
Cemiplimab in recurrent cervical cancer: Final analysis of overall survival in the phase III EMPOWER-Cervical 1/GOG-3016/ENGOT-cx9 trial (2025)	Phase III	United States, Australia, Brazil, Canada, Europe, Japan, Russia, South Korea, Taiwan, United Kingdom	Women with recurrent and/or persistent cervical cancer (n = 608)	Cemiplimab	Chemotherapy	Median OS: 11.7 months vs. 8.5 months	[25]
Durvalumab versus placebo with chemoradiotherapy for locally advanced cervical cancer (CALLA): a randomised, double-blind, phase 3 trial (2023)	Phase III, randomized, double-blind	Mexico, Peru, China, Europe, Mexico, Brazil, South Korea, India, Japan, Chile, Philippines, Russia, United States, Taiwan, South Africa	Women with previously untreated locally advanced cervical cancer (n = 770)	Durvalumab	Placebo	12-month PFS: 76.0% vs. 73.3%	[26]
Pembrolizumab or placebo with chemoradiotherapy followed by pembrolizumab or placebo for newly diagnosed, high-risk, locally advanced cervical cancer (ENGOT-cx11/GOG-3047/KEYNOTE-A18): overall survival results from a randomised, double-blind, placebo-controlled, phase 3 trial (2024)	Phase III, randomized, double-blind	Asia, Australia, Europe, North America, and South America	Women with newly diagnosed high-risk locally advanced cervical cancer (n = 1060)	Pembrolizumab–chemoradiotherapy	Placebo–chemoradiotherapy	36-month OS: 82.6% vs. 74.8%	[27]
Safety, efficacy, and immunogenicity of VGX-3100, a therapeutic synthetic DNA vaccine targeting HPV16/18 E6/E7 for CIN2/3 (2015)	Phase IIb, randomized, double-blind, placebo-controlled	USA, Estonia, South Africa, India, Canada, Australia, Georgia	Women aged 18–55 with HPV16/18-positive CIN2/3 (n = 167)	VGX-3100 DNA vaccine delivered by electroporation	Placebo	Histopathological regression to ≤CIN1 at 36 weeks: 49.5% vs. 30.6% (*p* = 0.034); HPV clearance with regression: 40.2% vs. 14.3%	[28]
A Phase II, Prospective, Randomized, Multicenter, Open-Label Study of GX-188E, an HPV DNA Vaccine, in Patients with Cervical Intraepithelial Neoplasia 3(2020)	Phase II, randomized, multicenter, open-label	South Korea	Women aged 19–50 with HPV16/18-positive CIN3 (n = 72)	GX-188E DNA vaccine via electroporation	NA	Histopathological regression: 52% at 20 weeks and 67% at 36 weeks	[29]
Regression of Human Papillomavirus Intraepithelial Lesions is Induced by MVA E2 Therapeutic Vaccine (2014)	Phase III, single arm	Mexico, Venezuela	Women with CIN 1, 2 or 3, or condyloma lesions Men with urethral condyloma or anal lesions	MVA E2	NA	Histological regression in 89% of women and 100% of men	[30]
Phase I and II randomized clinical trial of an oral therapeutic vaccine targeting human papillomavirus for treatment of cervical intraepithelial neoplasia 2 and 3 (2023)	Phase I/II, randomized, double-blind, placebo-controlled	Japan	Women with HPV-16-positive CIN2/3 (n = 165)	Oral Lactobacillus-based HPV-16 E7 vaccine (IGMKK16E7)	Placebo	Complete histological regression at 24 weeks: 31.7% vs. 12.5% (*p* = 0.05); strongest effect in HPV-16 only and CIN3 subgroups	[31]
Vvax001, a Therapeutic Vaccine, for Patients with HPV16-Positive High-grade Cervical Intraepithelial Neoplasia: A Phase II Trial (2025)	Phase II, open-label, single-arm	Netherlands	Women with newly diagnosed HPV16-positive CIN3 (n = 18)	Vvax001 (replication-deficient Semliki Forest virus vector encoding HPV16 E6/E7)	NA	Histopathological regression in 50%; lesion size reduction in 94%; HPV16 clearance in 63%	[32]

## Data Availability

No new data were created or analyzed in this study. Data sharing is not applicable to this article.

## Abbreviations

The following abbreviations are used in this manuscript:

ADCs	Antibody-Drug Conjugates
AIS	Adenocarcinoma In Situ
BFv	Bulumtatug Fuvedotin
CCRT	Concurrent Chemoradiotherapy (CCRT)
CIN	Cervical Intraepithelial Neoplasia
circRNA	Circular RNA
CRT	Chemoradiation
DAMPs	Damage-Associated Molecular Patterns
DCR	Disease Control Rate
DLT	Dose-limiting toxicities
E	Endostar
EMT	Epithelial–Mesenchymal Transition
FIGO	International Federation of Gynecology and Obstetrics (Fédération Internationale de Gynécologie et d’Obstétrique)
GI	Gastrointestinal
GU	Genitourinary
HIV	Human Immunodeficiency Virus
HPV	Human Papillomaviruses
HR	Hazard Ratio
hTERT	Human Telomerase Reverse Transcriptase
IFP	Interstitial Fluid Pressure
LACC	Locally Advanced Cervical Cancer
lncRNA	Long Non-Coding RNA
mAb	Monoclonal Antibody
MHC	Major Histocompatibility
miRNA	MicroRNA
ORR	Objective Response Rate
OS	Overall Survival
PFS	Progression-Free Survival
RCT	Randomized Controlled trial
SCC	Squamous Cell Carcinoma
TAMs	Tumor-Associated Macrophages
TF	Tissue Factor
TKI	Tyrosine Kinase Inhibitor
TPP1	Tripeptidyl Peptidase 1
TV	Tisotumab Vedotin
VEGF-A	Vascular Endothelial Growth Factor A
VEGFR	Vascular Endothelial Growth Factor Receptor
VLP	Virus-Like Particle
VN	Vascular Normalization
